# Epstein‒Barr virus and human herpesvirus 6 infection in patients with systemic lupus erythematosus

**DOI:** 10.1186/s12985-023-01987-3

**Published:** 2023-02-12

**Authors:** Xiaotong Chen, Hui Li, Chunling Wu, Yan Zhang

**Affiliations:** 1 The Department of Rheumatology and Immunology, The First Hospital of China Medical University, Shenyang, 110001 China; 2 Department of Pathogeny Biology, Basic Medicine College, Qingdao University, Qingdao, 266071 China; 3 Department of Clinical Laboratory, Zibo Central Hospital, Zibo, 255036 China; 4 Department of Clinical Laboratory, The Affiliated Hospital of Qingdao University, 19 Jiangsu Road, Qingdao, 266003 China

**Keywords:** Systemic lupus erythematosus, Epstein‒Barr virus, Human herpesvirus 6, Lytic gene, Latent gene

## Abstract

**Background:**

Systemic lupus erythematosus (SLE) is a complex autoimmune disease, and the etiology is still unclear. Some studies have indicated that viral infection might contribute to the development of SLE.

**Methods:**

A total of 105 individuals with SLE and 110 matched healthy controls were tested for EBV-specific DNA fragments in peripheral blood monocytes by PCR-Southern blotting. The expression of EBV-encoded genes was determined by RT-PCR and Southern blotting in EBV-positive patients. Serum EBV-specific IgM antibody was determined by ELISA. HHV-6 DNA in peripheral blood monocytes of those SLE patients and normal controls was tested by nested PCR.

**Results:**

Statistical analysis showed that the EBV-positive rate of SLE patients was significantly higher than that of the control group (χ^2^ = 87.329, *P* = 0), while the difference in the HHV-6-positive rate between the two groups was not significant (*P* > 0.05). An association of EBV and HHV-6 positivity in SLE patients was found (*P* = 0, *r* = 0.38). The EBV IgM level was significantly higher in SLE patients than in healthy controls (χ^2^ = 25.184, *P* = 0). Forty-two of the 75 EBV DNA-positive specimens were positive for EBNA2 mRNA, and an association between EBV EBNA2 mRNA and anti-Sm antibody positivity was found (*P* = 0, *r* = 0.409). LMP1 mRNA was positive in 2 SLE patients with active phase, and no LMP2A mRNA expression was detected in EBV DNA-positive specimens. EBV early gene BARF1 mRNA was detected in 2 cases of EBV-positive SLE patients, and these 2 patients were also HHV-6 DNA positive. Thirty-eight patients were BcLF1 mRNA positive, and 33 of them were HHV-6 positive as well. These factors were associated (χ^2^ = 15.734, *P* = 0). The expression of the EBV immediate early gene BZLF1 was negative in all 75 EBV-positive SLE patients.

**Conclusions:**

The results suggest that EBV infection might be related to the occurrence of SLE. Although there is no direct evidence that HHV-6 infection is associated with the development of SLE, EBV and HHV-6 infection may have a coacceleration effect in SLE patients. This study provides a new theoretical and experimental basis for the study of viral etiology and the prevention and treatment of SLE.

## Introduction

Systemic lupus erythematosus (SLE), which mainly occurs in women, is a complex autoimmune disease with a wide range of possible organ system injuries [1]. The characteristics of SLE are periodic flares and the production of autoantibodies against nuclear antigens, such as ribonucleoproteins (RNPs), Ro, and doble stranded DNA (dsDNA) [2]. Due to the complexity of SLE, the exact etiology and pathogenesis remain poorly understood, and most researchers believe that the etiology of SLE is multifactorial. Recently, infectious agents, including viruses, bacteria, and fungi, have been suggested to play important roles in the etiologies of SLE [3–5]. The potential mechanisms of infectious agents in the pathogenesis of SLE involve induction of aberrant innate and adaptive immunity, leading to a loss of tolerance to autoantigens. Some predominant pathogens have been reported to be associated with SLE [6], such as Epstein‒Barr virus (EBV) [7], human T-lymphotropic virus type 1 (HTLV-1) [8], and human immunodeficiency virus (HIV) [9].

Some immunochemical evidence suggests that molecular mimicry by infectious agents can active autoreactive T cells via exogenous antigens when these two antigens are sufficiently similar to one another to imitate or be a mimic. The antibody then disrupts the immune system and leads to an autoimmune response [10, 11]. Some studies have linked dysregulation of EBV to the development of SLE through several different lines of evidence. EBV nuclear antigen 1 (EBNA1) is the only protein required for maintenance of the viral genome. It was reported that there are at least three scenarios of autoimmunity being generated from the heteroimmune response against EBNA1, which cross-reacts with SLE-associated autoantigens, including Ro [12], Sm B/B′ [13], and Sm D1 [14]. Serological detection indicated that there is an association of the presence of anti-EBV-viral capsid antigen (VCA) and EBV DNA with SLE [15].

Human herpesvirus 6 (HHV-6) is a ubiquitous dsDNA virus that infects the majority of adults worldwide. HHV-6 has been less explored in the etiologies of SLE, and in previous serological studies, HHV-6 p41 was not examined in SLE patients [16]. However, Francesco Broccolo et al. reported that HHV-6 might act as a pathogenic factor predisposing patients to the development of autoimmune connective tissue diseases [17]. However, the causative role of HHV-6 in the development of SLE remains unclear. Both HHV-6 and EBV are herpesviruses. After primary infection, the latent virus can persist in the body for a long time, and the latent virus can be reactivated under the action of some factors. Recently, the interaction between viruses was reported to be one of the mechanisms of latent virus activation.

The aim of our study was to explore the role of EBV and HHV-6 infection in the occurrence and development of SLE, so as to provide new ideas for the etiological research and the prevention and treatment of SLE.

## Materials and methods

### Research object and ethical justification

SLE patient group: A total of 105 SLE patients were enrolled from the Department of Dermatology, Nephrology and Rheumatology of Affiliated Hospital of Medical College of Qingdao University, Department of Rheumatology of Qingdao Municipal Hospital. All patients met the 1982 American Rheumatology Association (ARA) diagnostic criteria for SLE [18]. There were 100 females and 5 males, aged 14–56 years. According to SLE disease activity index (SLEDAI) [19], SLE patients were divided into the active stage (≥ 9 was divided into active stage) and stable stage, with 55 patients in the active stage and 50 patients in the stable stage.

Control group: A total of 110 healthy subjects with corresponding sex and age were selected as the control group, with 98 females and 12 males, aged 14–61 years.

All applied protocols in this study, were in complete agreement with the Helsinki Declaration and its later amendment. This study received permission from the Medical Ethical Committee of the Medical College of Qingdao University. All patients signed written informed consent forms. For patients younger than 18 years old, their parents have signed the written informed consent forms.

### RNA and DNA extraction

Three milliliters of venous blood with EDTA were extracted, and the same amount of D-Hank’s solution was added and then centrifuged at 3000 r/min for 10 min at 4 °C. The supernatant was discarded, the precipitate was rinsed with D-Hank’s solution and centrifuged, this procedure was repeated 2–3 times, and the mononuclear cell suspension of the white membrane layer was aspirated. The mononuclear cell suspension was divided into two parts for DNA and RNA extraction, respectively. Total RNA was extracted using TRIzol (Invitrogen, USA) and was reverse transcribed using the First Strand cDNA synthesis kit (TaKaRa, Japan). The standard DNA extraction method with SDS-proteinase K digestion and phenol‒chloroform purification was used as described previously [20].

### Design and synthesis of primers and probes

The PCR primers and probes used to detect EBV and HHV6 genomes and mRNA expression of EBV-encoded genes were designed and synthesized by Beijing Cyparson Bioengineering Company. The sequences of primers and probes and the length of PCR products are shown in Tables 1 and 2. The Dig Oligonucleotide 3′-end Labeling Kit for oligonucleotide probes provided by Roche (Germany) was used to label oligonucleotide probes, and the labeling was performed in strict accordance with the instructions.

**Table 1:** Oligonucleotide primers and probes used for PCR

Transcripts	Primer sequences (5′-3′)	Product size (bp)
*EBV BamH I-W*		
5′ primer	CCAGACAGCAGCCAATTGTC	129
3′ primer	GGTAGAAGACCCCCTCTTAC	
Probe	CCCTGGTATAAAGTGGTCCTGCAGCTATTTCTGGTCGCATC	
*HHV 6 outer*		
5′ primer	AGTCATCACGATCGGCGTGTATC	287
3′ primer	TATCTAGCGCAATCGCTATGTCG	
*HHV 6 inner*		
5′ primer	TCGACTCTCACCCTACTGAACGAG	163
3′ primer T	GACTAGAGAGCGACAAATTGGAG

**Table 2:** Oligonucleotide primers and probes of EBV encoded genes used for RT-PCR analysis

Transcripts	Primer sequences (5′-3′)	Product size (bp)
*EBNA2*		
5′ primer C	GCTGCTACGCATTAGAGAC	339
3′ primer G	TCCTGGTAGGGATTCGAGG	
Probe	CAGCACTGGCGTGTGACGTGGTGTAAGTT	
*LMP1*		
5′ primer	TCCTCCTCTTGGCGCTACTG	490
3′ primer	TCATCACTGTGTCGTTGTCC	
Probe	GAACAGCACAATTCCAAGGAACAATGCCTG	
*LMP2A*		
5′ primer	ATGACTCATCTCAACACATA	280
3′ primer	CATGTTAGGCAAATTGCAAA	
Probe	ATCCAGTATGCCTGCCTGTA	
*BZLF1*		
5′ primer	CATGTTTCAACCGCTCCGACTGG	453
3′ primer ATG	GCGCAGCCTGTCATTTTCAG	
Probe	GCACGACGCACACGGAAACCACAACAGCCA	
*BcLF1*		
5′ primer	TGCCCAATCCCAAGTACACGACC	377
3′ primer	CAGCAGGTCATAATTGGACGGG	
Probe	GAGAGCATTCTGTAGGTTAAACGCGAGGAG	
*BARF1*		
5′ primer	GGCTGTCACCGCTTTCTTGG	203
3′ primer	AGGTGTTGGCACTTCTGTGG	
Probe	CTGGTTTAAACTGGGCCCAGGAGAGGAGCA	

### Southern blot

Digoxigenin-labeled oligonucleotide probes were added to the prehybridization solution (50% formamide, 5 × SSC, 5% dextran sulfate, 0.2%SDS, 1 × Denhardt’s solution, 25 mM sodium phosphate pH 7.4, 100 μg/ml salmon sperm DNA). The nylon membrane of transfer printing was placed in the hybridization bag, and the prehybridization solution (20 ml/100 cm^2^) was added at 42 °C for 1 h. Hybridization solution was added and hybridized overnight at 42 °C. The next day, the hybrid solution was discarded, and the membrane was washed successively with solution A (2 × SSC, 0.1% SDS) for 5 min × 2 times, solution B (0.5 × SSC, 0.1% SDS) for 15 min × 2 times, and solution C (2 × SSC, 0.3% Tween20) for 1–5 min. Blocking buffer was added for 30 min, add anti-digoxin antibody (1:5000) was added for 30 min, the membrane was washed with solution C for 15 min × 2, the membrane was washed with CSPD diluent for 2–5 min, and CSPD working solution was added to the film for 5 min. The sealed nylon membrane was placed in a dark box at 37 °C for 10 min to enhance the luminescence. After exposure for a certain time, the membrane was removed for development and observation.

### Detection of the BamH I-W fragment of EBV by PCR-Southern blot

The total volume of PCR was 25 μl, the reaction system was 10 × Buffer at 2.5 μl, 1.5 mmol/l MgCl_2_, 0.2 mmol/l dNTP, 0.5 μmmol/l upstream and downstream primers, 1.0 U Taq DNA polymerase, and 3 μl of DNA template. Positive and negative controls were established at the same time. The positive control was EBV-positive Akata cells, and the negative control was the EBV-negative Ramos cell line. The PCR was predenatured at 94 °C for 5 min. Then, 35 cycles of amplification were performed at 94 °C for 30 s, 55 °C for 30 s, and 72 °C for 45 s. Finally, the reaction was extended at 72 °C for 5 min. PCR amplification products were subjected to 2% agarose gel electrophoresis at 80 V for 1 h. Then, the membrane was transferred onto a Hybond-N^+^ nylon membrane and hybridized with a digoxin-labeled probe. The method was the same as above. Those with specific amplified bands at 129 bp were positive.

### Detection of EBV-encoded genes by PCR-Southern blot

Approximately 1 μg RNA of EBV-positive samples was subjected to cDNA synthesis with a reverse transcription system. The total volume of PCR reaction was 30 μl, the reaction system was 10 × Buffer at 3 μl, 1.5 mmol/l MgCl_2_, 0.1 mmol/l dNTP, 0.5 μmmol/l upstream and downstream primers, 1.0 U Taq DNA polymerase, and 3 μl cDNA template. Positive control and negative controls were set up at the same time. The positive control was EBV-positive LCL cells, and the negative control was the EBV-negative Ramos cell line. The PCR was predenatured at 94 °C for 5 min. Then, 35 cycles of 94 °C for 45 s, 58 °C for 45 s, and 72 °C for 1 min were used. Finally, the reaction was extended at 72 °C for 10 min. The PCR amplified products were electrophoresed and transferred to a membrane for hybridization. The method was the same as above.

### Nested PCR for HHV-6 detection

The amplification conditions were as follows: predenaturation at 94 °C for 5 min, denaturation at 94 °C for 30 s, renaturation at 58 °C for 30 s, and extension at 72 °C for 45 s for 35 cycles. Finally, the reaction was extended at 72 °C for 5 min. PCR products of the outer primers were used as templates for inner PCR amplification. The lateral PCR amplification product was 287 bp, and the medial PCR amplification product was 163 bp.

### Restriction analysis of HHV-6 PCR amplification products

The digestion reaction volume was 20 μl (2 μl of 10 × buffer, 10 μl of inner PCR product, 10 U of restriction endonuclease Hind III, and deionized water). The mixture was instantaneously centrifuged, and the reaction was performed in a 37 °C water bath for 12–16 h. Ten microliters of the enzyme digestion product was taken and subjected to vertical electrophoresis in a 12% polyacrylamide gel at 80 V for 1 h. Then the gel was removed and stained with EtBr (0.5 μg/ml) for 20 min. The electrophoresis results were observed and photographed under UV transmission instrument, and 66 bp and 97 bp bands were observed in HHV-6A, while HHV-6B was not digested. The PCR amplification band was still 163 bp.

### Serum EBV-specific IgM antibody was determined by ELISA

The serum specimen to be tested was diluted 1:100 with the specimen diluent. The samples were incubated at 37 °C for 40 min, washed with washing solution 3 times after removal, dried and added to the enzyme conjugate,and incubated at 37 °C for 20 min. Then, the samples were washed 3 times, substrate solutions A and B were added, and the samples were placed at room temperature for observation (5–10 min). Stop solution was added after color development of the positive control, 1 drop per well, and the results were observed.

### SLE anti-Sm antibody detection

The anti-Sm antibody was detected by an Anti-nuclear antibody spectrum (IgG) test kit (EUROIMMUN, Germany). According to the kit instructions, the serum was diluted with sample diluent and incubated with the test membrane for 30 min by shock. After washing, the enzyme conjugate was added and incubated for 30 min. After washing, substrate was added and incubated at room temperature for 10 min for observation.

### Statistical analysis

SPSS 11.0 was used to analyze the results of the experiment by the chi-square test, row × list chi-square test and four-square table exact test. *P* < 0.05 was considered significant.

## Results

### Detection of the EBV BamH I-W gene fragment

**Fig. 1:**
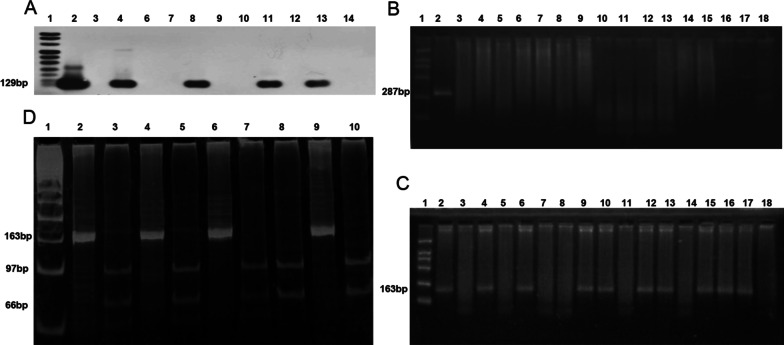
Detection of the EBV BamHI-W fragment and HHV 6. **A** Lane 1: DNA molecular weight marker VIII, DIG-labeled; Lane 2: Akata cells (positive control); Lane 3: Ramos cells (negative controls); other Lanes (4–14): SLE samples. **B** PCR results of HHV-6 lateral primers in some peripheral blood cell specimens. Lane 1: DNA marker DL2000; Lane 2: positive control (GS strain); Lane 3: negative control (J-Jhan cells); lane 4–18: PBMC specimens. **C** PCR results of HHV-6 inner primers in some peripheral blood cell specimens. Lane 1: DNA marker DL2000; Lane 2: positive control (GS strain); Lane 3: negative control(J-Jhan cells); Lane 4–18: PBMC specimens. **D** Electrophoretic diagram of PCR products digested inside some HHV6-positive specimens. Lane 1: 50 bp ladder DNA Marker; Lane 2–10: Results of medial PCR products of some HHV6 positive specimens digested with restriction endonuclease Hind III

PCR-Southern detection showed that the BamH I-W fragment of EBV was detected in 75 out of 105 SLE patients, with a positive rate of 71.43%. The BamH I-W fragment of the EBV genome was detected in 10 out of 110 normal controls, with a positive rate of 9.09%. The positive rate of EBV in SLE patients was significantly higher than that in the normal control group (χ^2^ = 87.329, *P* =  0) (Fig. 3A). Among them, 38 patients with active SLE were positive, the positive rate was 69.09% (38/55), which was significantly different from that of the control group (χ^2^ = 63.99, *P* = 0). Thirty-seven patients with stable SLE were positive, and the positive rate was 74.00% (37/50), which was significantly different from that of the control group (χ^2^ = 69.81, *P* = 0); the results are shown in Table 3. There was no significant difference in the EBV DNA detection rate between the active and stable SLE patients (χ^2^ = 0.309, *P* = 0.578). The length of the BamH I-W fragment amplified product was 129 bp, and the Southern hybridization results of some specimens are shown in Fig. 1A.

**Table 3:** The comparison of EBV DNA positivity in PBMCs between SLE patients and normal controls

Group	EBV ( +)	EBV (−)	Total	Positive rate (%)	value	χ^2^
SLE active	38	17	55	69.09	*P* = 0^a^	χ^2^ = 63.99^a^
SLE stable	37	13	50	74.00	*P* = 0^b^	χ^2^ = 69.81^b^
NC	10	100	110	9.09	*P* = 0^c^	χ^2^ = 87.329^c^

*NC* Normal control, *PBMC* Peripheral blood mononuclear cell

^a^Active compared with NC

^b^stable compared with NC

^c^SLE compared with NC

### HHV-6 DNA was detected by nested PCR

In 110 healthy control peripheral blood mononuclear cells (PBMCs), 64 samples were positive for HHV-6 DNA, the positive rate was 58.18% (64/110), and the length of the lateral PCR amplification product was 287 bp. In SLE patients, 56 cases were positive for HHV-6 DNA, and the positive rate was 53.33% (56/105) (Fig. 3B). No significant difference was found between the SLE and control groups (χ^2^ = 2.197, *P* = 0.138). The electrophoresis results of some specimens are shown in Fig. 1B. The length of the medial PCR amplification product was 163 bp. Figure 1C shows the medial PCR amplification results of HHV-6 in some peripheral blood cell specimens.

### Restriction digestion analysis of HHV-6 PCR products

The positive amplification product of HHV-6 (163 bp) was digested by the restriction endonuclease Hind III. After digestion of the HHV-6A PCR product, 66 bp and 97 bp bands could be observed, while HHV-6B was not digested by Hind III and remained as 163 bp inside the PCR amplification band. Both SLE patients and healthy controls showed the above two enzyme digestion results, namely, the HHV-6A and HHV-6B types were detected in both SLE patients. Among the 56 patients with HHV-6-positive SLE, 46 individuals (82.14%) were HHV-6B type, and 10 patients (17.86%) were HHV-6A type. In 64 cases of the HHV-6-positive normal control group, the HHV-6A type was 12.5% (8/64), and the HHV-6B type was 87.5% (56/64). The HHV-6 nested PCR detection results and HHV-6 restriction analysis results of some specimens are shown in Fig. 1D.

### Comparison of the EBV positive rate and HHV-6 positive rate in SLE patients

PCR results showed that the positive rates of EBV and HHV-6 were 71.43% (75/105) and 53.33% (56/105) in 105 SLE patients, respectively, and 49 of them had both EBV and HHV-6 gene fragments detected. EBV was positive in 10 of 110 healthy controls (9.09%, 10/110), and the HHV-6 gene fragment was detected in 64 cases (58.18%, 64/110), among which 2 cases were copositive. Statistical analysis showed that there was no significant difference in the positive rate of HHV-6 between the two groups. The positive rate of EBV in SLE patients was associated with the positive rate of HHV-6 (*P* = 0, r = 0.38). The results are shown in Table 4.

**Table 4:** The comparison between EBV positivity and HHV-6 positivity in SLE patients

EBV	HHV-6		Total
	+	−	
+	49	26	75
−	7	23	30
Total	56	49	105

χ^2^ = 15.188, *P* = 0, *r* = 0.38

### Expression of EBV latent genes

**Fig. 2:**
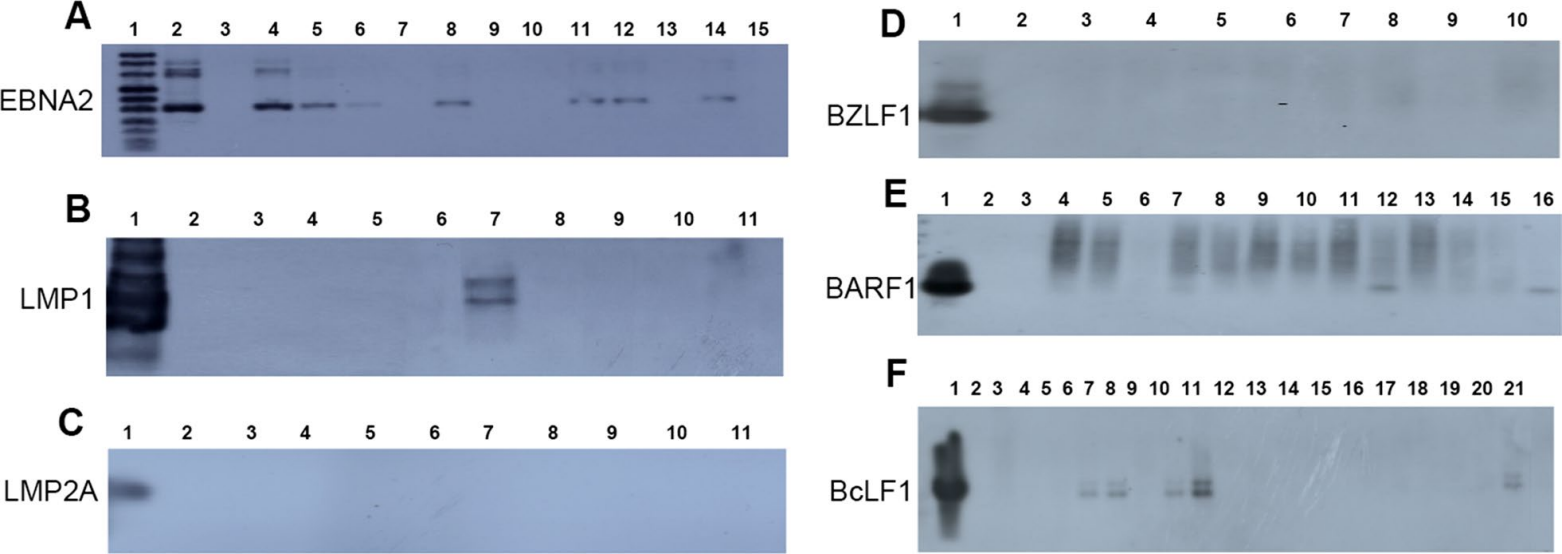
Detection of EBV latent and lytic genes expression. **A** PCR-Southern blotting of ths EBV latent gene EBNA2 in EBV-positive SLE samples. Lane 1: DNA molecular weight marker VIII, DIG-labeled; Lane 2: LCL(positive control); Lane 3: Ramos cells(negative controls); other Lanes: EBV-positive SLE samples. **B** and **C** PCR-Southern blotting of the EBV latent gene LMP1 and LMP2A in EBV-positive SLE samples. Lane 1: LCL (positive control); Lane 2: Ramos cells (negative controls); other Lanes: EBV-positive SLE samples. **D**–**F** RT-PCR-Southern blotting of EBV lytic gene BZLF1, BARF1 and BcLF1 in EBV-positive SLE samples. Lane1: LCL (positive control); Lane 2: Ramos cells (negative controls); other Lanes: EBV-positive SLE samples

The results of PCR-Southern hybridization showed that 42 of the 75 EBV DNA-positive specimens were positive for EBNA2 mRNA, 20 cases in the active phase and 22 cases in the stable phase. There was no significant difference in the positive rate between the active and stable phases (χ^2^ = 0.355, *P* = 0.551). LMP1 mRNA was positive in 2 patients in the active phase. There was no significant difference between the active phase and the stable phase (χ^2^ = 2.001, *P* = 0.493). No LMP2A mRNA expression was detected in 75 EBV DNA-positive specimens, and the results are shown in Tables 5 and 6. Figure 2A–C shows the detection results of EBNA2, LMP1 and LMP2A mRNA in some EBV-positive specimens.

**Table 5:** The comparison of EBNA2 mRNA positivity between SLE active phage and stale phage

Type	EBNA2 ( +)	EBNA2 (−)	Total (case)	Positive rate (%)	*P* value	χ^2^
SLE active	20	18	38	52.63		
SLE stable	22	15	37	59.46	0.551	0.355
Total	42	33	75	56.00

**Table 6:** The comparison of LMP1 mRNA positivity between SLE active phage and inactive phage

Type	LMP1( +)	LMP1 (−)	Total (case)	Positive rate (%)	*P* value	χ^2^
SLE active	2	36	38	5.26		
SLE stable	0	37	37	0.00	0.493	2.001
Total	2	73	75	2.67

### Expression of EBV genes in the lytic phase and its comparison with HHV-6 infection

The results of PCR-Southern hybridization showed that BARF1 mRNA was positive in 2 of the 75 EBV genome positive samples, both of which were active stage patients, and HHV-6 DNA was detected at the same time. There was an association between the BcLF1 mRNA detection rate and HHV-6 detection rate in EBV-positive SLE patients (χ^2^ = 15.734, *P* = 0). The results are shown in Table 7. No immediate early gene BZLF1 expression was detected in 75 EBV genome-positive specimens. Figure 2D–F shows the mRNA detection results of BZLF1, BARF1 and BcLF1 in some EBV-positive specimens.

**Table 7:** The comparison of positive rate between BcLF1 mRNA and HHV-6 DNA in EBV positive patient

HHV-6	BcLF1		Total
	+	−	
+	33	16	49
−	5	21	26
Total	38	37	75

χ^2^ = 15.734, *P* = 0, r = 0.458

### EBV-specific IgM antibody was determined by ELISA

**Fig. 3:**
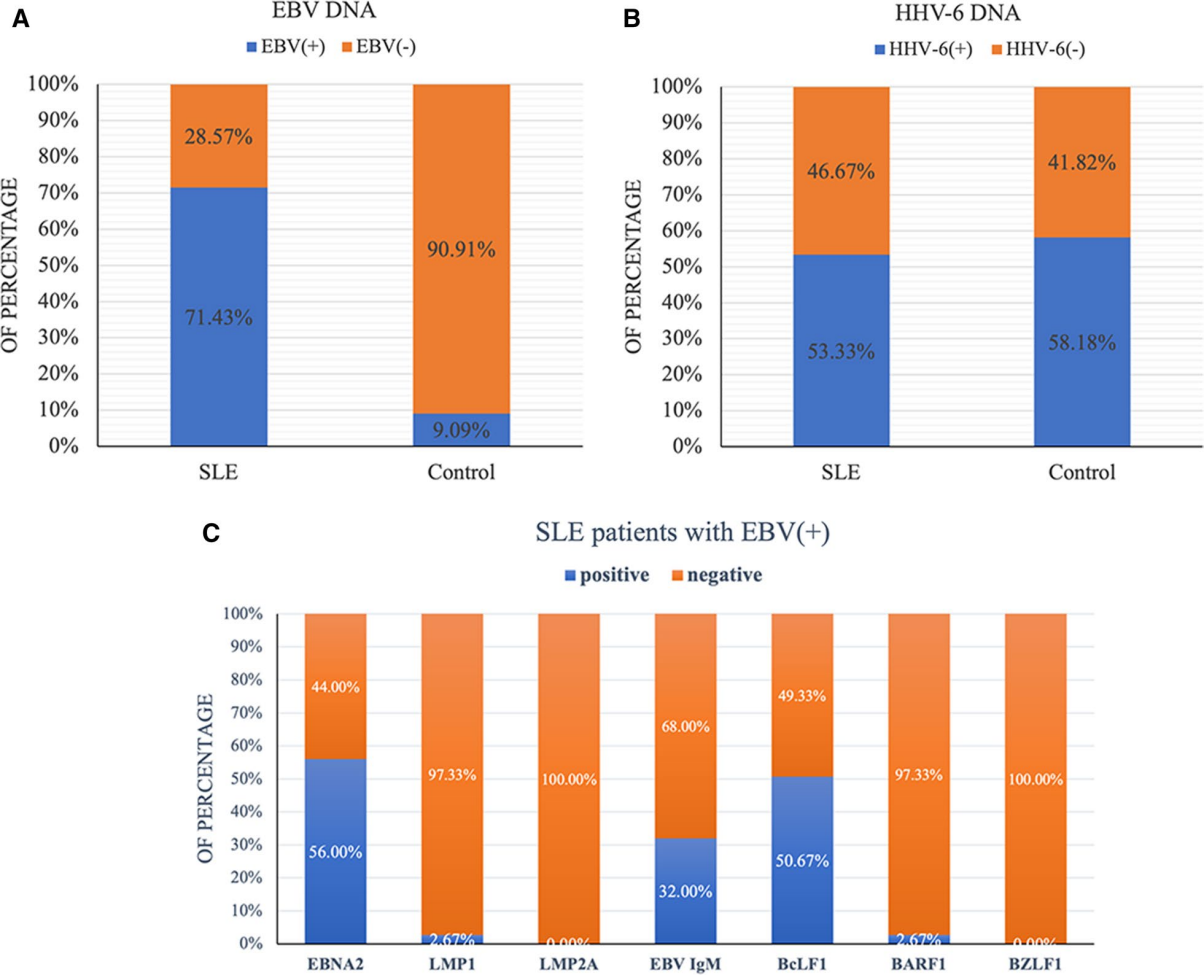
The positive proportion of EBV and HHV-6 in patients with SLE and healthy control. **A** The positive proportion of EBV DNA in patients with SLE and healthy control; **B** The positive proportion of HHV-6 DNA in patients with SLE and healthy control; **C** The positive proportion of EBV-encoded gene and EBV IgM antibody in EBV-positive SLE patients

ELISA was used to detect EBV-specific IgM antibodies in SLE patients and the controls. The results showed that 24 of 75 SLE patients were positive for EBV-specific IgM antibodies, with a positive rate of 32%. EBV-specific IgM antibody was positive in only 1 individual in the control group. The positive rate of SLE patients was significantly higher than that of the control group (χ^2^ = 25.184, *P* = 0). There was no significant difference in the detection rate of EBV IgM antibody between active and stable SLE patients (χ^2^ = 0.442, *P* = 0.506), among which 14 patients with active SLE were positive, and the positive rate was 25.45%, which was significantly different from that of the control group (χ^2^ = 26.73, *P* = 0). Ten patients with stable SLE were positive, with a positive rate of 20%, which was significantly different from that of the control group (χ^2^ = 19.568, *P* = 0). The results are shown in Table 8. Figure 3C shows the positive proportion of EBV-encoded gene and EBV IgM antibody in EBV-positive SLE patients.

**Table 8:** The comparison of EBV IgM between SLE patients and controls

Type	EBV IgM ( +)	EBV IgM (−)	Total (case)	Positive rate (%)
SLE active^a^	14	41	55	25.45
SLE stable^b^	10	40	50	20.00
NC^c^	1	109	110	0.91

^a^SLE active type; ^b^SLE stable type; ^c^normal control. SLE(a + b) compared with NC, χ^2^ = 25.184, *P* = 0; SLE(a) compared with SLE(b), χ^2^ = 0.442, *P* = 0.506; SLE(a) compared with NC, χ^2^ = 26.73, *P* = 0; SLE(b) compared with NC, χ^2^ = 19.568, *P* = 0

### Comparison of the expression of EBNA2 mRNA and anti-Sm antibody in SLE patients

Among the SLE patients, 40 were positive for anti-Sm antibody. Among the 75 EBV positive samples, 42 were positive for EBNA2. Among the 40 SLE patients with positive serum anti-Sm antibody, 30 were positive for EBNA2 mRNA, and among the 35 SLE patients with negative serum anti-Sm antibody, 12 were positive for EBNA2 mRNA. The association between the detection rate of serum anti-Sm antibody and EBNA2 mRNA in SLE patients is shown in Table 9. Statistical analysis showed that the detection rate of anti-Sm antibody was associated with the detection rate of EBNA2 (*P* = 0, r = 0.409).

**Table 9:** The related analysis between positivity of anti-Sm antibody and EBNA2 mRNA expression in SLE patients

Anti-Sm	EBNA2 mRNA		Total
	+	−	
+	30	10	40
−	12	23	35
Total	42	33	75

χ^2^ = 12.558, r = 0.409, *P* = 0

**Fig. 4:**
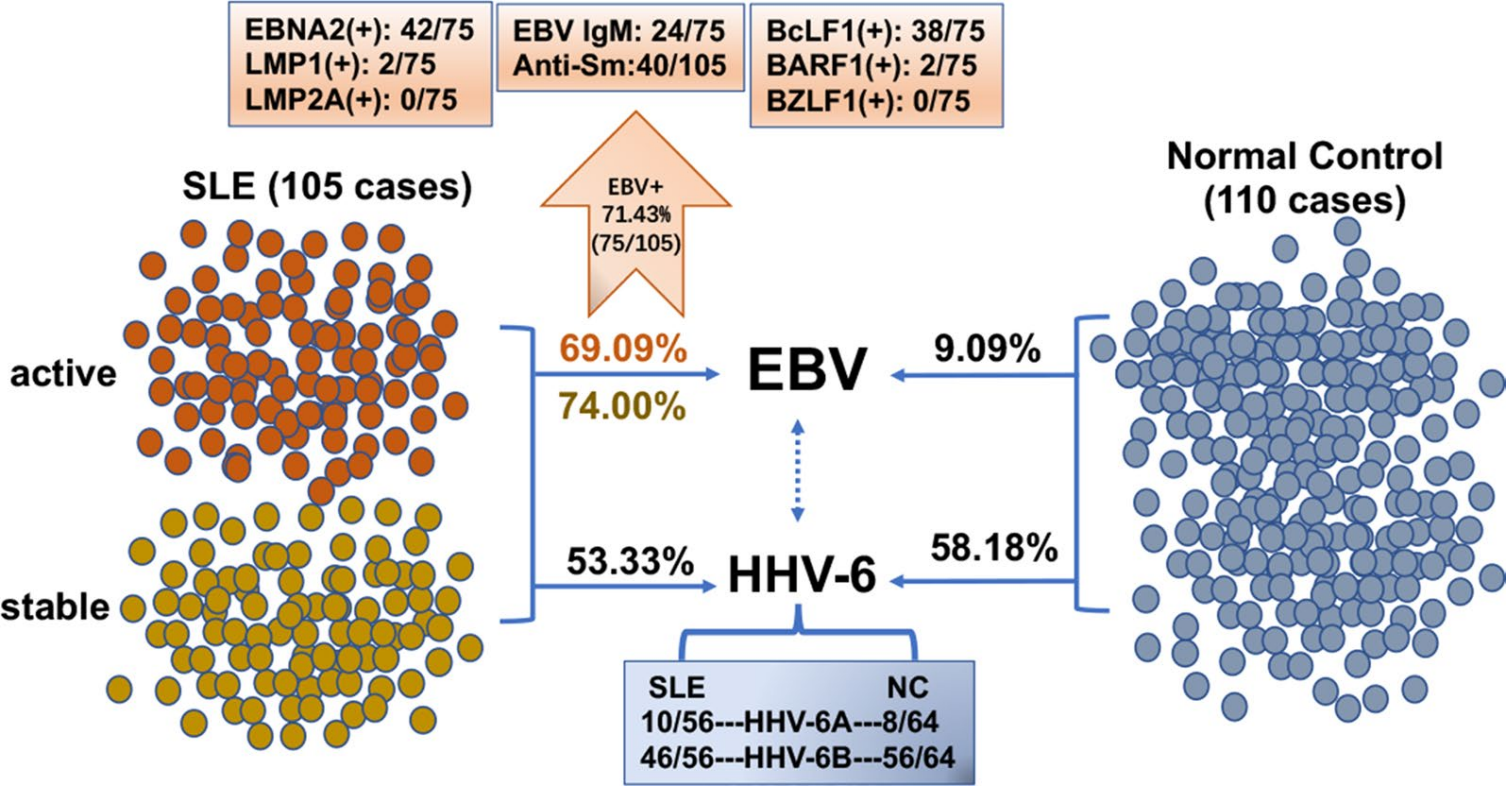
EBV and HHV-6 infection in patients with SLE and healthy control. The positive rate of EBV was 71.43% (75/105) in SLE patients, with the active type accounting for 69.09% and the stable type accounting for 74.00%. In the controls, it was only 9.09% (10/110). In 75 SLE patients who were EBV positive, 42 cases were EBNA2 positive, 2 cases were LMP1 positive, 38 cases were BcLF1 positive, 2 cases were BARF1, and 24 cases were EBV IgM positive, while LMP2A and BZLF1 mRNA was not detected. The positive rate of HHV-6 was 53.33% (56/105) in SLE patients, and 58.18% (64/110) in controls. In 56 HHV-6-positive SLE patients, 10 cases of HHV-6A and 46 cases of HHV-6B were detected. In 64 healthy controls, 8 cases of HHV-6A and 56 cases of HHV-6B were detected

In summary, Fig. 4 summarizes the main findings of this study for EBV and HHV-6 infection in patients with SLE and healthy control.

## Discussion

SLE is a complex nonorgan-specific autoimmune disease that can affect multiple organs, such as the skin, joints, kidney, and heart. At present, there is no therapeutic drug with clear efficacy. The typical course of the disease is demonstrated by periods of disease flares alternating with remission. SLE mainly affects young women (90% of cases), and the prominent characteristics of SLE are a large number of immunoactive autoantibodies, activation of polyclonal B lymphocytes and production of inflammatory cytokines in the patient’s serum [21, 22]. Environmental factors play an important role in the pathogenesis of SLE. However, to date, no specific environmental pathogenic factors have been found in epidemiological studies, and different pathogenic factors may induce SLE in different individuals. In recent years, the viral etiology of SLE has received increased attention, and become a research hot spot [23, 24]. An in-depth study of the relationship between viral infection and the pathogenesis of SLE is of great significance to better understand the etiology and pathogenesis of SLE and to find new treatment methods.

EBV is the causative agent of infectious mononucleosis (IM). It is a B lymphocytophilic agent. EBV infects mature human B lymphocytes by binding to CD21 on the surface of B lymphocytes and entering the infected cells through receptor-mediated endocytosis. EBV infection may induce autoimmunity [23, 25]. In recent years, attention has been given to the relationship between EBV infection and SLE. An elevated level of EBV DNA was found in 42% of SLE patients but in only 3% of healthy controls. The findings indicated that the reaction of EBV is associated with the development of SLE and flares [26]. Several research teams have demonstrated that SLE patients have high expression of viral mRNAs, such as BZLF, gp350, LMP1, LMP2, and EBNA1, while little or no mRNA was observed in normal immune competent carriers of EBV [27, 28]. In contrast to previous studies, we described in detail the expression of EBV DNA, IgM antibody, and latent and lytic genes encoded by EBV in peripheral blood. We also detected HHV-6 detection and its association with EBV. We found that the positive rate of EBV DNA and specific IgM antibody in SLE patients was significantly higher than that in healthy controls, suggesting that EBV primary or active infection was associated with the pathogenesis of some SLE patients. This finding is consistent with previous studies.

EBV infection usually is divided into latent and lytic phases. In the latent infection phase, the expression of EBV products is limited. While in the lytic phase, the virus replicates in large numbers. EBV-positive specimens were further detected for the expression of the latent genes EBNA2, LMP1, and LMP2A and the lytic genes BZLF1, BARF1 and BcLF1. EBNA2 is encoded by BYRF1 with a molecular weight of approximately 81–85 kD. It is a necessary protein for transformation and can interfere with B-cell metabolism and transform B cells [29]. Incaprera et al. found that a high degree of homology between the EBNA-2 354GRGKGKSRDKQRKPGGPWRP373 subsequence and the antigenic C-terminal domain 101GRGRGRGRGRGRGRGGPRR119 of the SmD1 ribonucleoprotein, a target of autoantibodies in a portion of SLE patients [30]. In this study, 42 of the 75 EBV (+) SLE specimens were positive for EBNA2 mRNA. There was an association between the detection rate of serum anti-Sm antibody and the detection rate of EBNA2 mRNA in EBV-positive SLE patients, while there was no significant difference in the positive rate between active and stable phase patients. These results indicated that EBNA2 might be related to the pathogenesis of SLE and could induce immune dysfunction in SLE patients. LMP1 is a functional protein that binds to cell membranes and plays a certain role in maintaining B-cell activation, transformation, and immune regulation. EBV LMP1 was reported to increase SLE-related autoantibodies in mice [31]. In this study, there were only 2 cases with LMP1 mRNA positivity in the SLE active stage. Due to a variety of factors, such as technical methods and number of specimens, the correlation between LMP1 mRNA expression and the occurrence and development of SLE needs to be further studied. The results of Miller et al. suggested the LMP2A could block the Ca^2+^ channel of B cells and inhibit the virus from entering the proliferation cycle, thus maintaining the latent state of EBV infection [32]. LMP2A allows anti-Sm B cells to overcome the regulatory checkpoint at the early preplasma cell stage by a self-Ag-dependent mechanism [33]. The results of this experiment showed that LMP2A expression was not detected in SLE specimens with EBV DNA positivity. The loss of LMP2A expression in EBV DNA positive SLE specimens suggested that EBV could not block the Ca^2+^ channel of B cells through LMP2A in EBV-positive SLE patients, and then inhibited the virus from entering the proliferation cycle. Therefore, we speculated that the loss of LMP2A expression is related to the infection of EBV-positive SLE patients in the lytic phase.

EBV immediate early genes are the earliest genes expressed after the viral genome is active from the latent to the lytic phase. BZLF1 expression controls the switch from the latent to lytic phase. The EBV early gene BARF1 encoding product is related to cell transformation function, which can transform primary monkey renal epithelial cells and human epithelial cells in vitro [34]. PCR-Southern hybridization showed that 75 EBV DNA positive patients were negative for BZLF1, and 2 patients were positive for BARF1 mRNA, all of whom were active stage patients. The late EBV gene BcLF1 mainly encodes the viral capsid antigen (VCA). In the process of EBV proliferation, progeny virions are assembled in the nucleus. VCA and EBV DNA assemble into viral nucleocapsids. Two meta-analyses have confirmed that EBNA1 and VCA IgG had a strong association between EBV seropositivity and SLE [35, 36]. In this study, 38 of the 75 EBV-positive SLE patients were positive for BcLF1 mRNA expression, indicating that the expression of BcLF1 mRNA may be related to the pathogenesis and progression of SLE.

Recently, the interaction between viruses was reported to be one of the mechanisms of latent virus reaction. Studies have suggested that HHV-6 can co-cause host coinfection with other viruses, such as EBV and HCMV. Due to the increase in HHV-6 binding sites on the surface of EBV-infected B lymphocytes, proteins including EBV receptors on the surface of infected cells are also increased, which can activate latent EBV to the lytic infection phase, leading to the polyclonal activation of B lymphocytes [37]. Tagawa et al. [38] found that EBV could activate the latent HHV-6 in vivo and increase its corresponding antibody titer by more than four times. HHV-6 can also infect cells carrying the EBV genome and promote EBV replication. Flamand et al. [39] showed that HHV-6 replication directly induced EBV proliferation cycle, and multiple genes of HHV-6 could transactivate EBV in B lymphocytes, increasing the expression of the EBV immediate early antigen Zebra and early antigen (EA-D and EA-R) up to 10 times. In addition, after HHV-6 infection, the expression of EBV late gene products increased making EBV transition from a latent state to a lytic state, and leading to the occurrence of EBV-related diseases. Although the results of this study showed that there was no significant difference in the positive rate of HHV-6 in SLE patients compared with healthy controls, and no direct evidence of HHV-6 infection related to the occurrence of SLE was obtained. The results of this study also showed that the detection rate of EBV in SLE patients was associated with the detection rate of HHV-6. Therefore, the possibility of HHV-6 combined with EBV coinfection in SLE patients was increased. This result suggested that the synergistic effect of HHV-6 and EBV might lead to the activation of polyclonal B lymphocytes and abnormal immune function.

Our study has several limitations. Due to the limitations of time and objective conditions, the number of SLE patients was not sufficiently large, and the study failed to separate in children and adolescents from adult with SLE. The EBV infection rate of children and adolescents is lower than that of adults, so the conclusions may be different. The relationship between EBV infection status and pathological changes in SLE patients and how the virus enters the active lytic phase from the latent state need to be further studied.

## Conclusion

In conclusion, our results suggested a strong association of EBV infection with SLE. Although HHV-6 infection does not seem to be associated with this disease, but there was an association between the EBV detection rate and HHV-6 detection rate in SLE patients. Further study on the relationship between EBV infection, viral lytic latency gene expression and the pathogenesis and progression of SLE is important for revealing the causal relationship between EBV infection and SLE and exploring new methods for the prevention and treatment of SLE.

## Data Availability

The datasets used and/or analyzed during the current study are available from the corresponding author on reasonable request.

## Abbreviations

SLE: Systemic lupus erythematosus
EBV: Epstein–Barr virus
HHV-6: Human herpesvirus 6
RNPs: Ribonucleoproteins
dsDNA: Doble stranded DNA
HTLV-1: Human T-lymphotropic virus type 1
HIV: Human immunodeficiency virus
EBNA1: EBV nuclear antigen 1
VCA: Viral capsid antigen
ARA: American Rheumatology Association
SLEDAI: SLE disease activity index
PBMC: Peripheral blood mononuclear cell
IM: Infectious mononucleosis
